# *Pseudomonas fluorescens* imparts cadmium stress tolerance in *Arabidopsis thaliana* via induction of AtPCR2 gene expression

**DOI:** 10.1186/s43141-022-00457-7

**Published:** 2023-01-25

**Authors:** Chinreddy Subramanyam Reddy, Min Cho, Tanushri Kaul, Jin Tae Joeng, Kang Min Kim

**Affiliations:** 1 Division of Biotechnology, College of Environmental and Bio Resource Science, Chonbuk National University, Iskan, 570-572 Republic of Korea; 2 Department of Biology, West Virginia State University, 141 Hamblin Hall, Institute, WV 25112-1000 USA; 3 Nutritional Crop Improvements, International Center for Genetic Engineering and Biotechnology, New Delhi, India; 4 Medicinal Crops Division, National Institute of Horticultural and Herbal Science, Rural Development Administration, Eumseong, Chungbuk 27709 Republic of Korea

**Keywords:** Cadmium stress, PCR2, *Pseudomonas*, Transgenics, *Arabidopsis*

## Abstract

**Background:**

Cadmium is a non-essential, third largest heavy metal contaminant with long retention time that poses environmental hazards. It emanating majorly from industrial processes and phosphate fertilizers. Cadmium is effortlessly assimilated by plants and leads to yield loss. Henceforth, identification of mechanisms to attenuate the heavy metal toxicity in crops is beneficial for enhanced yields.

**Results:**

Beneficial soil bacteria have been known to combat both biotic and abiotic stress, thereby promoting plant growth. Amongst them, *Pseudomonas fluorescens* has been shown to enhance abiotic stress resistance in umpteen crops for instance maize and groundnut. Here, we investigated the role of *P. fluorescens* in conferring cadmium stress resistance in *Arabidopsis thaliana.* In silico analysis of PCR2 gene and promoter revealed the role, in cadmium stress resistance of *A. thaliana*. Real-time expression analysis employing qRT-PCR ratified the upregulation of *AtPCR2* transcript under cadmium stress up to 6 folds. Total leaf (50%), biomass (23%), chlorophyll content (chlorophyll-a and b 40%, and 36 %) silique number (50%), and other growth parameters significantly improved on bacterial treatment of the 2mM Cd-stressed plants.

**Conclusion:**

Moreover, generated 35s-promoter driven *AtPCR2* over-expressing transgenic lines that exhibited resistance to cadmium and other heavy metal stress. Taken together, a crucial interplay of *P. fluorscens* mediated enhanced expression of *AtPCR2* significantly induced cadmium stress resistance in *Arabidopsis* plants.

**Supplementary Information:**

The online version contains supplementary material available at 10.1186/s43141-022-00457-7.

## Background

Cadmium (Cd) is a non-essential, toxic heavy metal, enormously increasing in the environment due to industrialization. It is one of the global major pollutants, and it has highly toxic nature due to reactivity with sulfhydryl groups, which may inhibit the enzymatic activities [1]. Cd significantly inhibits the plant growth and biomass by interrupting water, mineral uptake, and photosynthesis [2]. It negatively impacts photosynthesis via disorganization of grana, thereby leading to reduction in chlorophyll biosynthesis [3]. Cd is toxic for plants even at low concentrations, while concentrations higher than 5–10 μg Cd g^-1^ leaf dry weight are lethal to plants [4]. In nature, plants exhibit an innate tolerance to Cd stress to a certain extent; however, at elevated Cd concentrations, improved growth and crop yields may only be garnered via employing novel strategies to withstand Cd or heavy metal toxicity for sustainable agriculture development.

Mutual interactions between beneficial mycorrhizae helper bacteria and plants have been reported to accelerate crop growth and yields. Recent studies revealed that bacterial interactions contributed to increased biotic and abiotic stress resistance in plants [5–10]. *Pseudomonas fluorescens* is a non-toxic, ecofriendly, and widely distributed rhizo-bacterium, which releases beneficial phyto-hormones, anti-microbial substances, metal chelators, and volatile organic compounds (VOCs) as well as excretes hydrolytic enzymes, such as proteases, cellulose, chitinase, and β-glucanase [11, 12]. This potent diversity of compounds directly or indirectly promotes growth, in addition to biotic and abiotic stress resistance in umpteen crops. Multiple reports exhibited the protective role of *P. fluorescens* in conferring stress resistance [12–14]. Conjointly, around 25 volatile bacterial odors elicited have been identified, which were correlated to numerous differentially expressing plant mRNAs related to stress responsiveness, hormonal regulation, and cell metabolism. Previously, Wang and coworkers [15] performed microarray analysis of *Arabidopsis thaliana* treated with *P. fluorescens* and revealed an upregulation in 95 genes at the transcript level. Amongst them, the plant cadmium resistance 2 gene (*PCR2*) exhibited increased (5.4-fold) transcript levels corresponding to heavy metal tolerance. This motivated us to elucidate the potential impact of *Arabidopsis-Pseudomonas* interaction on the degree of Cd resistance within the host plant.

The present investigation was undertaken in order to reveal the role of *P. flourescens* on *Arabidopsis* plant growth and heavy metal (Cd) tolerance. To validate the *Arabidopsis thaliana PCR2* (*AtPCR2*) expression, we performed qRT-PCR in accordance to [15]. Invariably, *AtPCR2* gene expression was monitored in *P. fluorescens* treated *A. thaliana* interaction studies during Cd stress. Further, to corroborate the findings from qRT-PCR of *AtPCR2*, in silico analysis of its promoter region was performed to highlight its role in Cd stress resistance. Moreover, we assayed the biomass, chlorophyll content, leaf, and silique numbers of *A. thaliana* Cd stressed lines that we primed with *P. fluorescens* in comparison to control lines (in the absence of bacterial treatment). To elucidate the underlying molecular mechanism, we have generated *AtPCR2* transgenic lines employing floral dip method. Overexpression studies of *PCR2* gene in Cd-stressed *A. thaliana* lines would mimic the *P. fluorescens* treatment and enable us to obtain insights into the mechanism of Cd-stress resistance. Insights into the influence of *P. fluorescens* on plant growth and heavy metal resistance in *Arabidopsis* may be extrapolated to other crops for modulating both productivity and stress resistance.

## Methods

### Bacterial suspension cultures

*P. fluorescens* KACC10327 was streaked onto Luria Bertani (LB) agar plates and grown at 28+1°C in dark for 24h. Single discrete colony was picked from LB agar plate using sterile tooth pick and transferred into liquid LB media followed by incubation at 28+1 °C (250 rpm) to yield 10^-9^ml^−1^colony-forming units (CFU), as calculated by optical density (OD) and serial dilution counts [6].

### AtPCR2 transcript analysis in *Arabidopsis thaliana* treated with P. fluorescens

Plants were kept in growth chamber enabled with 16/8h light (with 120–150 μmol/m^2^ s) and dark photoperiod. Healthy growing 14 days old plants were subjected with *P. fluorescens* cells with 50 μl of (1×10^8^ bacteria/ml) simultaneously control plant was treated with LB media. The total RNA was isolated from *P. fluorescens* treated *A*. *thaliana* ecotype Col-0 seedlings at various time points, i.e., 0, 3, 6, and 12 h post-inoculation. The total RNA was isolated by TRIZOL method (Invitrogen, San Diego, CA) according to manufacturer’s instructions. cDNA synthesis was performed using mRNA (1.0 μg) employing an ImProm-II first-strand cDNA synthesis system (Promega Corp., Madison, WI) with an oligo(dT)18 primer. Real-time qRT-PCR was performed using SYBR Green PCR Master Mix (Applied Biosystems, San Diego, CA) and an ABI Step One Plus thermocycler (Applied Biosystems). Cycling conditions were maintained as follows; 95°C for 10 min, followed by 40 cycles of 95°C for 30 s, 52°C for 25 s, and 72°C for 30 s. Each experiment was performed in triplicate with three independent replicates. The RT-PCR amplification and quantification of the expression of *AtPCR2* gene (NM_001332138.1) transcript levels was normalized against *Actin2* gene (NM_001338358.1) as an internal control. The list of primers used in this study is mentioned in the Table 1.

**Table 1:** Primers used in this study listed in the table

Gene	Primer	Product size (bp)
*AtPCR2* F(Full length)	5′-TACAAAACCTTACATTGCTTTC-3′	859
*AtPCR2* R(Full length)	5′-TATTGTTTTGGTGTCACTTTCT-3′	
*AtPCR2* F(RT-PCR)	5′-GGTCCACAGGCTTCTGTGAT-3′	99
*AtPCR2* R(RT-PCR)	5′-CTACAATCTCGGCGACTTGG-3′	
*Actin*-2 F	5′-AGT GGT CGT ACA ACC GGT ATT GT-3′	92
*Actin*-2 R	5′-GAT GGC ATG AGG AAG AGA GAA AC-3′	

### In silico analysis of promoter

Upstream genomic sequences (∼1 kb from transcription start) of *Arabidopsis thaliana PCR2* gene were retrieved from TAIR9. The cis-regulatory element analysis was performed using http://www.bioinformatics2.wsu.edu/cgi-bin/Athena/cgi/visualize.pl. Identified elements were highlighted with different colors in the Fig. 2

### Expression analysis of PCR2 gene using micro array data and co-expression

The expression data of *Arabidopsis PCR2* genes were retrieved from the AtGenExpress (http://jsp.weigelworld.org/expviz/expviz.jsp) under various stresses as described previously [16]. The log2-transformed values were used to generate heat maps, and hierarchical clustering was performed using MeV software [17]. *AtPCR2* gene regulatory network was identified by co-expression analysis tool http://genecat.mpg.de/cgi-bin/Ainitiator.py

### Plant growth materials and treatments

*A. thaliana* (ecotype Col-0) seeds were initially surface sterilized with 70% ethanol for 5 min, followed by 2% sodium hypochlorite (NaOCl) for 10 min, and rinsed with sterile distilled or (milliQ) water about ten times. Seeds were germinated on MS media (half-strength) and 7-day-old seedlings of uniform growth were carefully transferred to pre-sterilized plastic pots (diameter 10cm, depth 8cm, one seedling per pot) containing 100g of heat-sterilized (95°C, 36 h) vermiculite and soil mix (1:1) supplied with Hoagland’s nutrient solution [25 mL of half strength; KNO_3_(5mM), (NH)_4_H_2_PO_4_ (1mM), Ca(NO_3_)_2_ (0.5mM), MgSO_4_ (0.5mM), Fe-Citrate (60μM), H_3_BO_3_(92μM), MnCl_2_·4H_2_O (18μM), ZnSO_4_·7H_2_O (1.6μM), CuSO_4_·5H_2_O (0.6μM), and (NH _4_)_6_Mo7O_24_·4H_2_O (0.7 μM)] once a week. After 1 week, the soil was treated with bacterial suspension culture (10-^9^ cfu/ml in 1m LB) and soil inoculated with only liquid LB medium (1mL) was used as control. In case of administration of cadmium stress treatments, seedlings were supplied with nutrient solution supplemented with CdCl_2_ (1, 2 mM). The experiments were replicated in triplicate. *Arabidopsis* plants were maintained under controlled conditions at 23+1°C in the growth chamber.

### Quantification of plant growth parameters

We employed *Arabidopsis* plants (30 days old) for determination of plant growth and physiological index measurements. Total plant leaf and silique numbers were counted. Plants were gently detached from the pots and fresh root and shoot weights, root length, and shoot height were measured. Leaf chlorophyll content was estimated according to [18]. Fresh leaf samples were ground thoroughly with acetone (80%) in the dark and centrifuged at 9000 g for 10 min at 4°C. Absorbance was measured at 645 and 663nm for collected supernatant on UV-2102C Spectrophotometer, Unico Instrument Co., Ltd, Shanghai, China.

### Statistical analysis

Data generated by assaying of parameters for instance growth; physiological index, transgenic analysis, and so on were compiled in the form of mean standard deviations (*n*=6). Statistical analyses, one-way ANOVA, and Duncan’s multiple range tests were performed.

### Cloning of AtPCR2 cDNA and plant transformation

The *AtPCR2* cDNA expression cassette that comprised of 35S CaMV Promoter: *AtPCR2* cDNA:NOS-terminator was cloned into pCB302ES plant transformation vector harbouring BASTA resistant gene “bar” as the selection marker and hemagglutinin (HA) epitope tag [19]. Further, the recombinant plant transformation vector harbouring the *AtPCR2* expression construct was transformed into *Arabidopsis thaliana* employing the agrobacterium (GV3101)-mediated floral-dip method [20]. We generated putative T_0_, T_1_, T_2_, and T_3_ transgenic lines that were screened by PCR analyses employing genes specific primer for *AtPCR2* and BAR. In order to perform stress experiments, T_3_ homozygous lines harboring *AtPCR2* were used for further experiments.

### Protein extraction and immunoblot analysis of AtPCR2 transgenic lines

Total solubilized rosette leaf proteins were extracted from transgenic and wild type lines using extraction buffer [SDS (2%), TRIS (pH 6.8; 60mM), β-mercaptoethanol (14.4mM), glycerol (10%), and bromophenol blue (0.1% (w/v)]. Total protein concentration was determined using Bio-Rad protein assay reagent (Bio-Rad Laboratories, Hercules, CA, USA) and BSA (bovine serum albumin; Kit II, Catalog No. 500-0002) as standard. Protein extracts (50 μg) were fractionated on a 10% SDS–PAGE and electro-blotted onto a nitrocellulose PVDF membrane (Hybond-C membrane; Amersham Biosciences, Piscataway, NJ, USA) according to [21]. *AtPCR2*-HA proteins were detected using anti-HA-HRP IgG antibodies (Cell Signaling, Hitchin, UK) and visualized using a Super Signal® West Femto maximum sensitivity substrate kit (Thermo Scientific, Waltham, USA).

### Stress analysis of transgenic lines

Sterilized seeds of transgenic and WT lines were germinated on MS (half-strength) media. Seedlings (7 days old) were transferred to fresh MS (half-strength) media supplemented with various abiotic stressors for instance; salt (NaCl; 150 mM), heavy metal (CdCl_2_; 50 μM), cold (only MS media; 4°C), and (80μM CuSO4).

## Results

### P. fluorescens-induced AtPCR2 transcript expression confers Cd tolerance

**Fig. 1:**
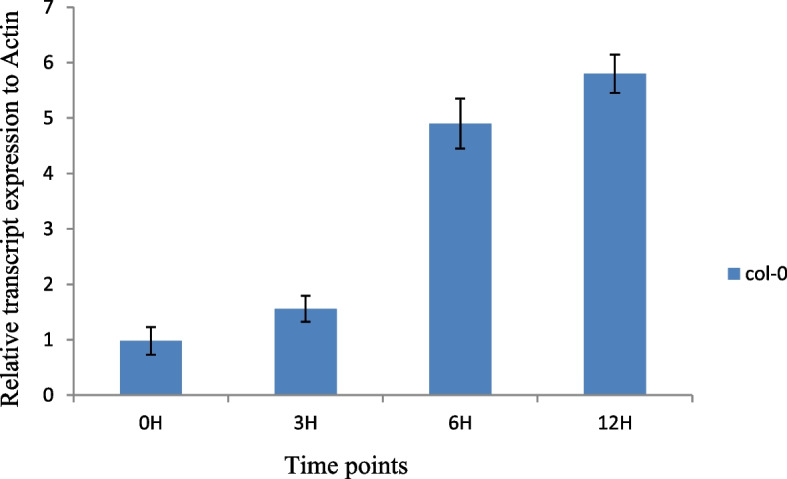
Relative *AtPCR2* m-RNA expression level while *Pseudomonas fluorescens* and *Arabidopsis thaliana interaction*. Bars indicate standard deviation (*n* = 3). Statistically significant differences (Student’s *t* test) between transgenic lines and Col-0 were indicated (*p* < 0.05)

The qRT-PCR was carried out to quantify the real-time expression profiles of *AtPCR2* transcript in *Arabidopsis* lines that were treated with *P. fluorescens* for varying time periods in comparison to WT. The *AtPCR2* transcript abundance was normalized against that of internal control actin. We found that *AtPCR2* transcript levels increased in a time-dependent manner, i.e., *AtPCR2* transcript levels enhanced by 1.75-, 4.8- and 6-folds after 3h, 6h, and 12h interaction with *P. fluorscens*, respectively (Fig. 1). Results strongly suggested that the enhanced expression of*AtPCR2* gene in *Arabidopsis* lines on interaction with *P. fluorscens* had a definite functional role in conferring cadmium tolerance to them.

### In silico analysis of promoter

**Fig. 2:**
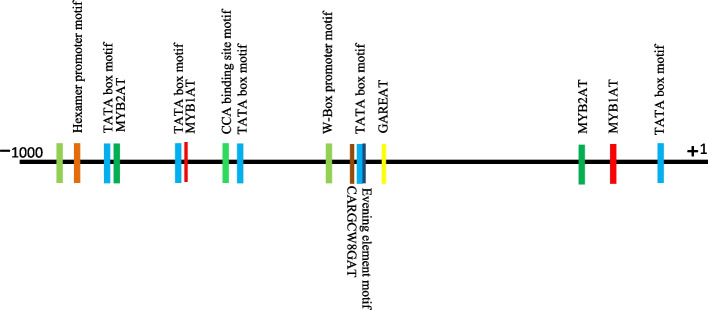
Putative cis-regulatory elements identified in the promoter regions of *PCR2* gene from *Arabidopsis thaliana*. The approximate positions of putative cis-regulatory elements were predicted in the 1 kb upstream region of the *PCR2* gene by PlantCARE database

In order to unveil the transcriptional regulation of *AtPCR2* gene in *Arabidopsis*, we retrieved the 1 kb upstream regions or putative promoter sequences and analyzed them using PlantCARE. A number of important cis-regulatory elements and stress-responsive motifs were identified (Fig. 2). A few known stress-related cis-regulatory elements (CREs) involved in stress responsive expressions for instance-dehydration responsive MYB1AT and transcriptional activator of ABA (MYB2AT), cold-response element (Evening Element), Gibberellin responsive element (GARE), phytochrome-modulated (CCA1), and wound-responsive WRKY (W-Box) family elements were revealed in the putative promoter region of *AtPCR2* gene. The presence of these stress-related motifs in the upstream promoter region of *AtPCR2* gene may be directly correlated to the altered gene expression on interaction with *P. fluorscens*.

### Microarray data and co-expression analysis

In response to stress, *AtPCR2* gene expression was high in roots, whereas mild expression was detected in shoots. Interestingly, we noted that *AtPCR2* expression was highly upregulated in response to cold, salt, oxidative stress, and UVB exposure in the roots across different time periods within 24 h (Supplementary Figures S1 and S2).

### *Pseudomonas fluorescens* boosted *Arabidopsis* growth during Cd stress

**Fig. 3:**
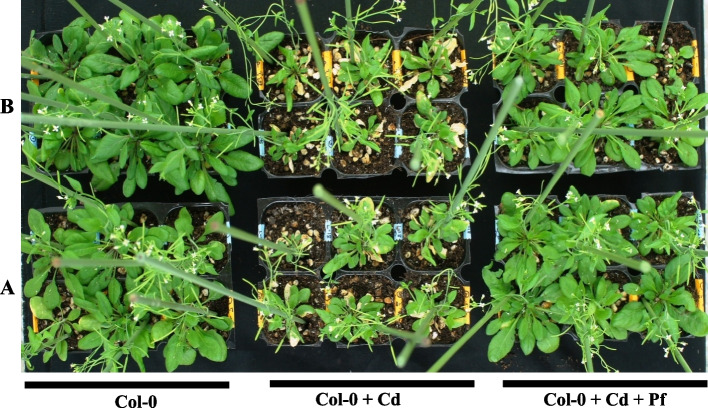
Col-0: Columbia-0 *Arabidopsis* line. **a** Control. **b** *Pseudomonas fluorescens* treated. Col-0 + Cd: Columbia-0 plants with cadmium stress. **a** 1mM. **b** 2mM. Col-0 + Cd + Pf: In the presence of *Pseudomonas fluorescens* cadmium treatment to *Arabidopsis* plants. **a** 1mM and **b** 2mM

**Fig. 4:**
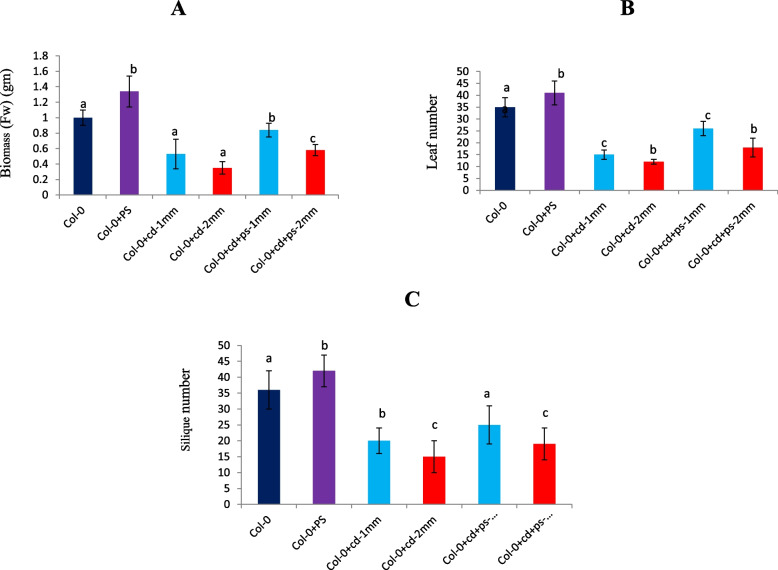
Effects of *Pseudomonas fluorescens* on total biomass, leaf, and silique number of *Arabidopsis* under different CdCl2 concentrations. Values are means and bars indicate SDs (*n*=6) columns with **a**, **b**, and **c** letters indicate statistically significant difference at *P*< 0.05 (Duncan test)

**Fig. 5:**
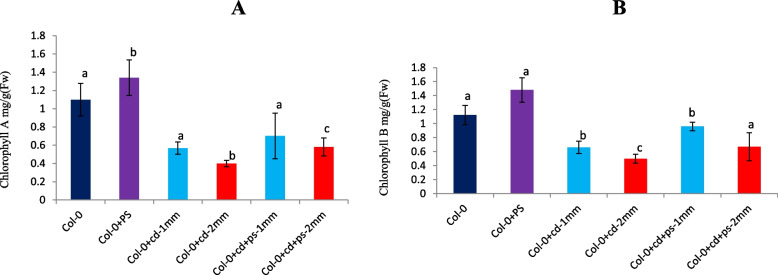
Effects of *P. fluorescens* on chlorophyll a and b content of *Arabidopsis* under different CdCl2 concentrations. Values are means and bars indicate SDs (*n*=6) columns with **a**, **b**, and **c** letters indicate statistically significant difference at *P*< 0.05 (Duncan test)

*P. fluorescens* interaction led to enhanced growth of *A. thaliana*, even under cadmium stress (Fig. 3). Total plant biomass (fresh weight) increased by about 25%, with *P. fluorescens* treatment in comparison to untreated WT during normal conditions. However, during *P. fluorescens* treatment in combination with Cd stress (1mM, 2 mM), the plant biomass revealed further enhancement by 58 and 23 %, respectively, in comparison to stand alone Cd treatment (Fig. 4A). Leaf and silique number showed an increment of 69%, 50%, and 41.3%, 30.56%, during combinatorial treatment of cadmium stress (1mM and 2mM, respectively) along with the presence of *P. fluorescens*; in comparison to only Cd treatment (Fig. 4B, C). *P. fluorescens* significantly improved chlorophyll a and b content in *Arabidopsis thaliana* by 25% and 40% (*P* <0.01) and 45 % (*P* <0.05), 36 % (*P* <0.01) during cadmium treatments 1mM and 2mM, respectively (Fig. 5).

### Transgenic *Arabidopsis thaliana* lines expressing AtPCR2

**Fig. 6:**
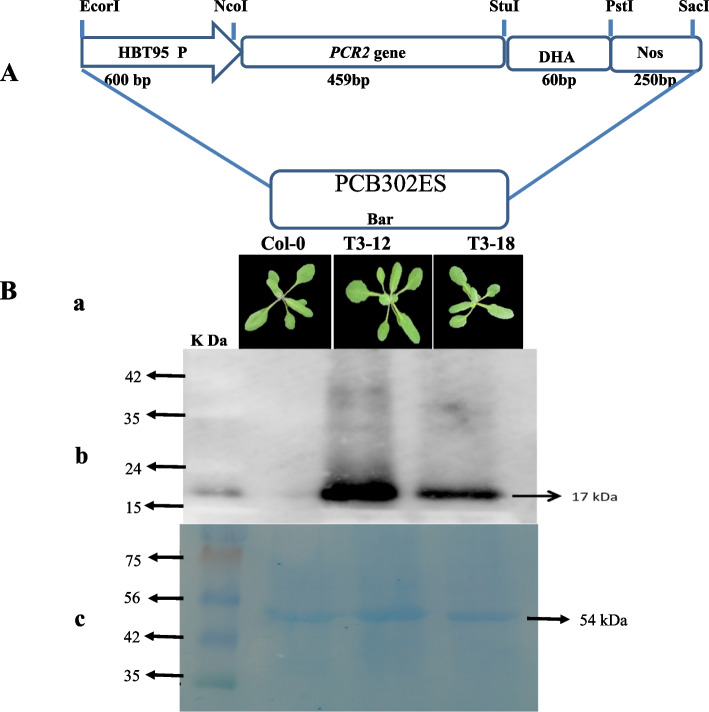
**A** Plant transformation vector construction with restriction sites. **B a** Col-0 and transgenic lines (Col-0, T3-12, and T3-18) grown at normal conditions; **b** Lane B contains Western blot of Col-0, T3-12, and T3-18 (overexpressed PCR2 protein-detected 17kDa with HA-tagged antibodies); **c** Line C contains SDS-PAGE of the Col-0, T3-12, and T3-18 (Rubisco protein 54 kDa)

To investigate the function of *AtPCR2* in effectuating plant resistance responses against cadmium stress, we generated transgenic *Arabidopsis* lines over expressing *AtPCR2* gene and selected two independent T_3_ homozygous lines; T_3_-12 and T_3_-18. Under normal growth condition, both line T_3_-12 and T_3_-18 exhibited similar growth and developmental patterns to WT (Figs. 6Aa, S3). Among selected transgenic lines, line T_3_-12 exhibited higher expression level of *AtPCR2* than line T_3_-18 (Fig. 6Bb). Further, *AtPCR2*-HA proteins were detected in transgenic lines. Among transgenic lines, the accumulation of *AtPCR2*-HA was higher in T_3_-12 than T_3_-18. We detected the accumulation of *AtPCR2*-HA protein in the T_3_-12 and T_3_-18 transgenic *Arabidopsis* plants, whereas no *AtPCR2*-HA protein was accumulated in WT (Fig. 6b). The rubisco protein was used as a reference (54KD) (Fig. 6c) protein which is abundantly present in the universe).

### Overexpression of AtPCR2 confers resistance against stress

**Fig. 7:**
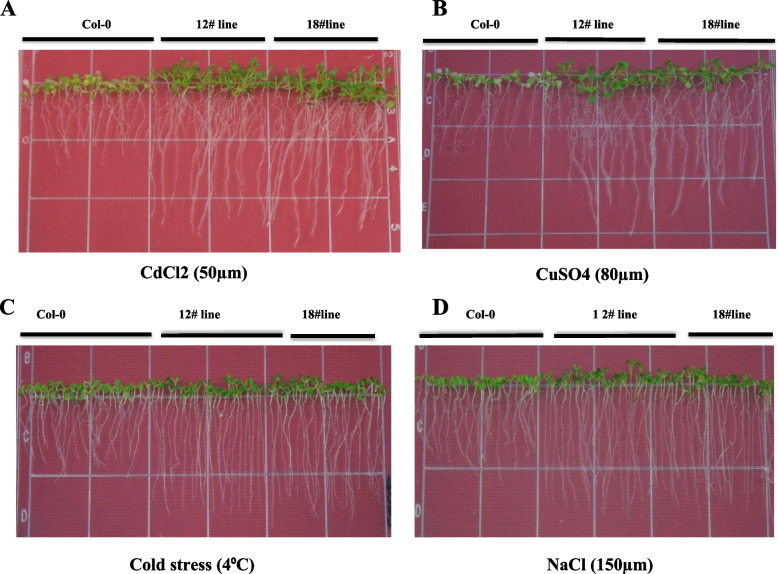
Various abiotic stresses effect on AtPCR2 transgenic lines: **A** Heavy metal (50-μM cadmium chloride) treatment. **B** An 80-μM CuSO4 heavy metal treatment. **C** Cold stress treatment (4°C). **D** Salt stress (150-mM sodium chloride). Col-0, columbia-0 control; AtPCR2 line, transgenic *Arabidopsis* line

In a bid to investigate the role of *AtPCR2* gene over expression in conferring stress resistance to *A. thaliana*, we exposed both transgenic and WT lines to various abiotic stresses. *AtPCR2* over-expressing *A. thaliana* lines showed significantly enhanced resistance various stresses for instance; cadmium chloride (CdCl_2_), copper sulphate (CuSO_4_), sodium chloride (NaCl), and cold in comparison to WT. T_3_-12 line exhibited higher levels of stress resistance than T_3_-18 line (Figs. 7, S4).

## Discussion

Previously, it has been elucidated that beneficial soil bacteria promote growth of umpteen plant species [10, 22, 23]. Beneficent soil bacteria-mediated plant growth promotion induced during abiotic stress conditions has been observed in several cultivated and wild plant species including tomato (*Solanum lycopersicum*) [24], chickpea (*Cicer arietinum*) [25], and alfalfa (*Medicago sativa*) [26]. Numerous studies revealed that plant-growth promoting rhizobacteria (PGPR) improve plant growth and yield during adverse ambient conditions via production of a spectrum of siderophores, ACC-dehydrogenase, microbial-emitted volatiles (mVOCs), or chemicals and other hormone-modulated compounds. Hormone-modulated compounds trigger the differential expression of numerous transcripts involved in cell-wall modifications, primary and secondary metabolisms, stress responses, hormone regulation, and protein expression in *Arabidopsis* [16]. Similarly, Zhang and colleagues [6] have shown PGPR GB03-induced HKT1 gene expression augments salt resistance. A recent study has revealed that *Azotobacter vinelandii* strain SRI Az3 aids to combat chromium stress tolerance in rice plants via significantly boosting its antioxidant machinery [27].

The present study focuses on the underlying mechanisms of *PCR2* upregulation and its role in imparting cadmium stress resistance to *Arabidopsis*. We elucidated the direct correlation between *Pseudomonas* triggered *PCR2* gene induction and its contribution towards abiotic stress tolerance in *Arabidopsis* plants. Invariably, it provides direct evidence for high transcript induction of *AtPCR2* that up-regulates by 5.4 folds within a 12-h span of interaction between *Arabidopsis* (host) and *Pseudomonas* (Fig. 1). In line with our results, Wang et al. data [16] demonstrate similar *PCR2* transcript induction that peaks (six-fold) at 12h time-point, post interaction with *Pseudomonas*. In silico investigations of cis-regulatory elements in the upstream region of *PCR2* gene reveals crucial stress regulatory elements for instances cold responsive MYB1, MYB2, gibberellin responsive GARE and salicylic acid responsive, and wound responsive WRKY family W-box (Fig. 2). *PCR2* co-expression analysis suggests that it is closely related tothe PLAC8 family of proteins (At4g23470, At1g52200) and *AtPCR1*, which play imperative roles in determining fruit size and heavy metal transportation [1] (Fig. S1). Microarray expression analysis across multiple abiotic stress treatments reveals differential expression of *PCR2* with abundance in roots as compared to aerial parts (Fig. S2). In silico analysis exhibits numerous resident cis-acting elements in the upstream promoter region of *PCR2* that evidently displays its crucial involvement in combating abiotic stress.

During stress conditions, enhanced growth of *Arabidopsis* plants directly correlates to the presence of *Pseudomonas* in soil. Our results are consistent with previous reports demonstrating that *Pseudomonas* inoculation in soil significantly increases stress tolerance in plants [13, 14]. Previous studies suggest that *P. fluorescens* can tolerate high concentrations of Cd [28]. Moreover, a strain of *P. aeruginosa* performs as an efficient Cd accumulator [29]. Reports are consistent with our analysis of improved Cd tolerance levels in *A. thaliana* (up to ~0.75mM) on interaction with *P. fluorescens*. Further, an exposure of *Arabidopsis* plants to CdCl_2_ (1–2 mM) results in significantly high levels of tolerance to Cd along with growth enhancements in comparison to WT (Fig. 3).

Leaf development plays a crucial role in plant survival and growth, since it affects the area available for photosynthesis, which in turn corresponds positively to plant biomass accumulation [30, 31]. In the current study, *Pseudomonas* inoculation significantly increases the total biomass, silique-, and leaf number per plant, in *Arabidopsis* (Fig. 4). In addition, leaf chlorophyll content is an important physiological trait that directly correlates to photosynthetic rate in plants [32]. Previous studies show that plants grown under Cd stress, synthesize less chlorophyll, abnormal grana structure, and low dry matter than those without Cd exposure [3]. Several studies confirm that PGPRs augment photosynthetic capacity by increasing photosynthetic efficiency and chlorophyll content on exposure to abiotic stress [6, 10, 33]. In our investigation, *Pseudomonas* also remarkably enhances leaf chlorophyll content and biomass on exposure to Cd stress conditions (1 and 2 mM CdCl_2_) (Fig. 5). In context with this, *PCR2* over-expressing transgenic T_3_ lines under CaMV35 promoter (Fig. 6B) exhibit higher accumulation of PCR2 protein than WT along with robust stress tolerance (Fig. 7A–D). Among the 10 isoforms of *PCR* gene family, the *PCR2* has a vital role in heavy metal detoxification in yeast as well as in *Arabidopsis* [34]. Taken together, our results elucidate the efficacy of *P. fluorscen*s in imparting heavy metal stress tolerance via inducing high expression of *AtPCR2* gene. Conclusively, our results offer in-depth insights into the possible mechanisms of PGPR-*Pseudomonas* in combating cadmium stress in plants.

## Conclusion

Although we proposed that *P. fluorscen*s in imparting heavy metal stress tolerance via inducing high expression of *AtPCR2* gene, complete molecular mechanism is yet to be elucidated to describe how it is mitigating other abiotic stress conditions.

## Supplementary Information

**Additional file 1: Fig. S1.** *PCR2* gene regulatory network analyzed by co-expression analysis.

**Additional file 2: Fig. S2.** Expression analysis of *Arabidopsis PCR2* gene responses todifferentstress conditions in the shoot and root tissues. Microarray expression data for *AtPCR2* gene was retrieved from TAIR (ver 9) during various abiotic stresses, i.e. salt, drought, osmotic, cold, heat, oxidative, genotoxic, wounding and UV/B stress. The datasets obtained for various time points of stress, namely 0.5, 1, 3, 6, 12 and 24 h, were analyzed with respect to the control. The colour bar below represents relative expression values; wherein green represents lowest, black represents medium and red signifies highest expression levels. The hierarchical clustering is performed, and heat maps have been generated using TIGR MeV software package.

**Additional file 3: Fig. S3.** Controlled condition grown Col-0, T3-12, T3-18 *Arabidopsis thaliana* plants.

**Additional file 4: Fig. S4.** Various abiotic stress conditions imposed to control vs overexpressed lines and their root length and biomass data. Values are means and bars indicate SDs (*n*=6) statistically significant difference at *P*< 0.05 (t-test).

## Data Availability

The datasets used and/or analyzed during the current study are available from the corresponding author on reasonable request.

## Abbreviations

AtPCR2: *Arabidopsis thaliana* plant cadmium resistance-2 gene
Cd: Cadmium
mM: Milli molar
