# Supplementary figures and images for: Pseudomonas fluorescens imparts cadmium stress tolerance in Arabidopsis thaliana via induction of AtPCR2 gene expression
Source: J Genet Eng Biotechnol. 2023 Jan 25;21:8. doi: 10.1186/s43141-022-00457-7 (PMC9877264; doi:10.1186/s43141-022-00457-7)

## Slide 1
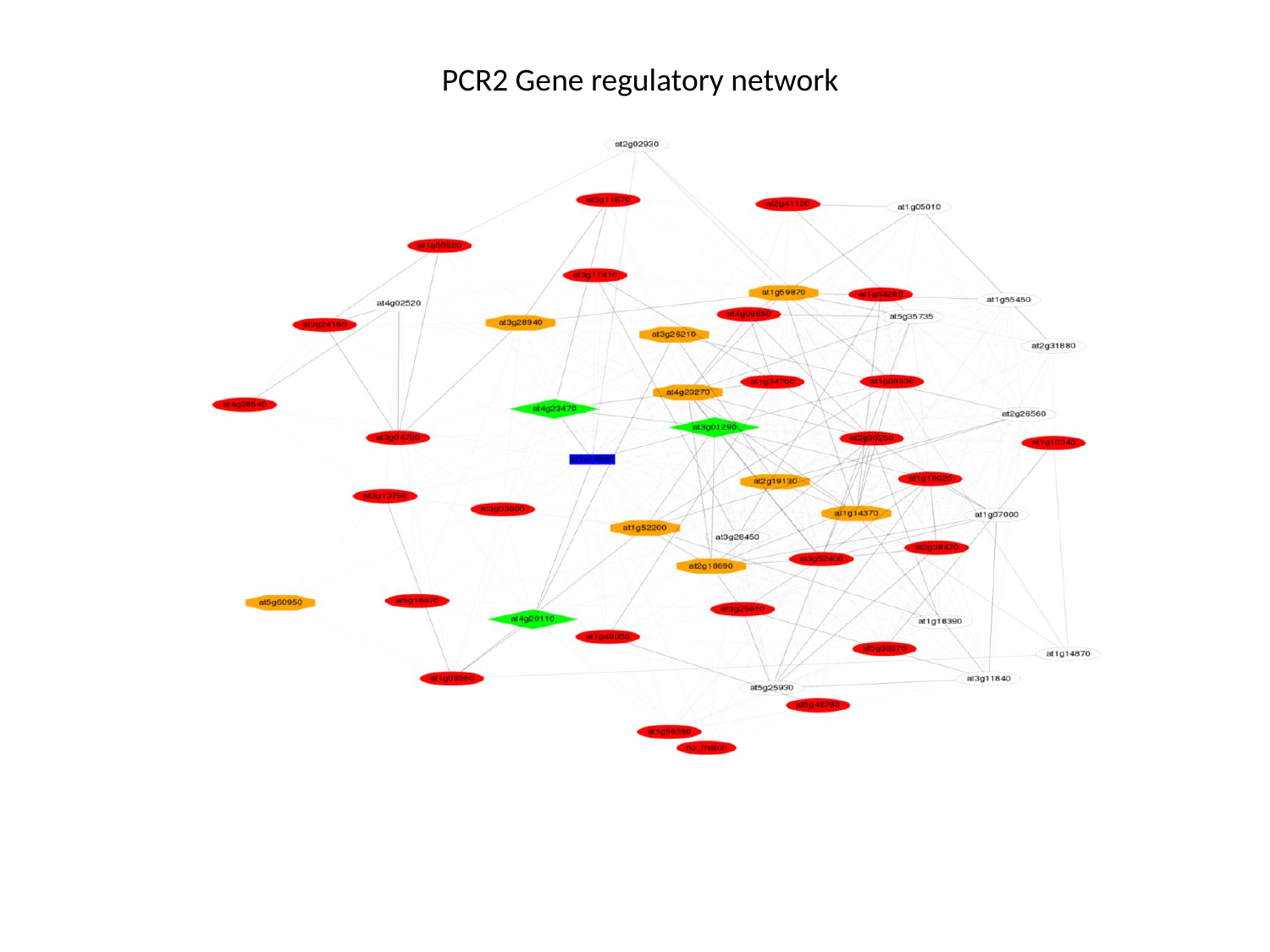

PCR2 Gene regulatory network

Supplement: Supplementary file 1 — Additional file 1: Fig. S1. PCR2 gene regulatory network analyzed by co-expression analysis. [file 43141_2022_457_MOESM1_ESM.ppt]

## Slide 1
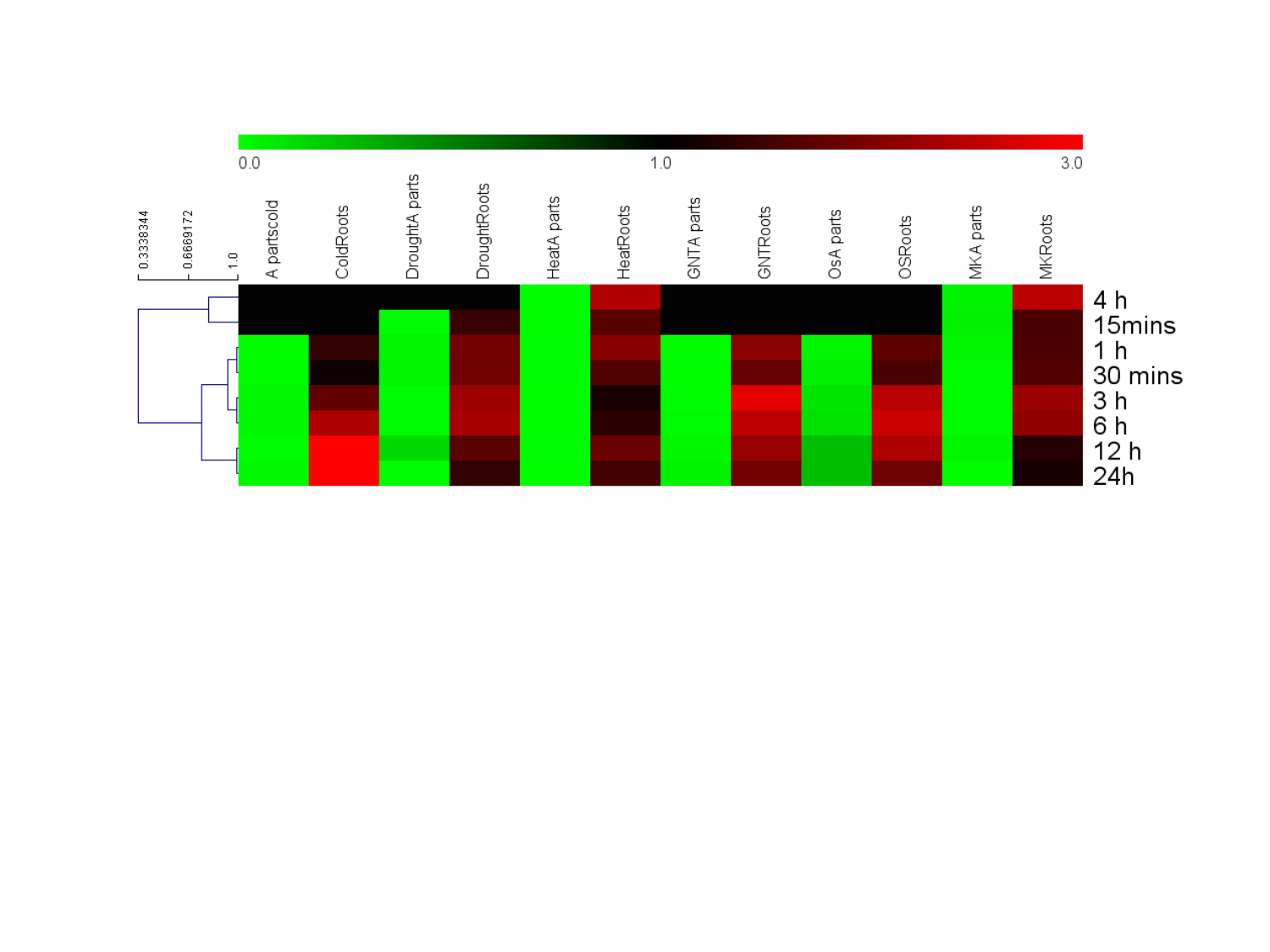

Supplement: Supplementary file 2 — Additional file 2: Fig. S2. Expression analysis of Arabidopsis PCR2 gene responses todifferentstress conditions in the shoot and root tissues. Microarray expression data for AtPCR2 gene was retrieved from TAIR (ver 9) during various abiotic stresses, i.e. salt, drought, osmotic, cold, heat, oxidative, genotoxic, wounding and UV/B stress. The datasets obtained for various time points of stress, namely 0.5, 1, 3, 6, 12 and 24 h, were analyzed with respect to the control. The colour bar below represents relative expression values; wherein green represents lowest, black represents medium and red signifies highest expression levels. The hierarchical clustering is performed, and heat maps have been generated using TIGR MeV software package. [file 43141_2022_457_MOESM2_ESM.ppt]

## Slide 1
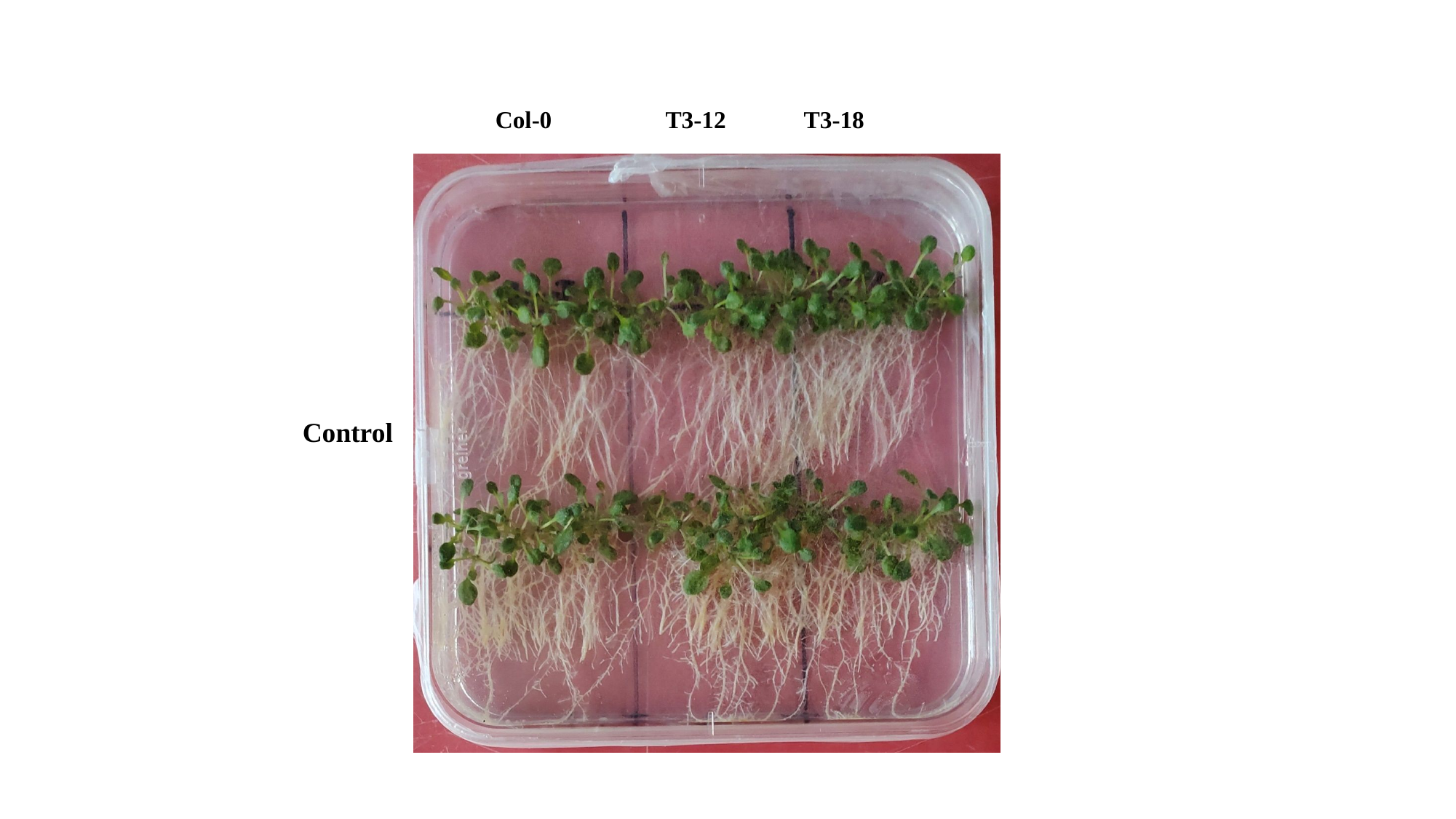

Col-0 T3-12 T3-18
Control

Supplement: Supplementary file 3 — Additional file 3: Fig. S3. Controlled condition grown Col-0, T3-12, T3-18 Arabidopsis thaliana plants. [file 43141_2022_457_MOESM3_ESM.ppt]

## Slide 1
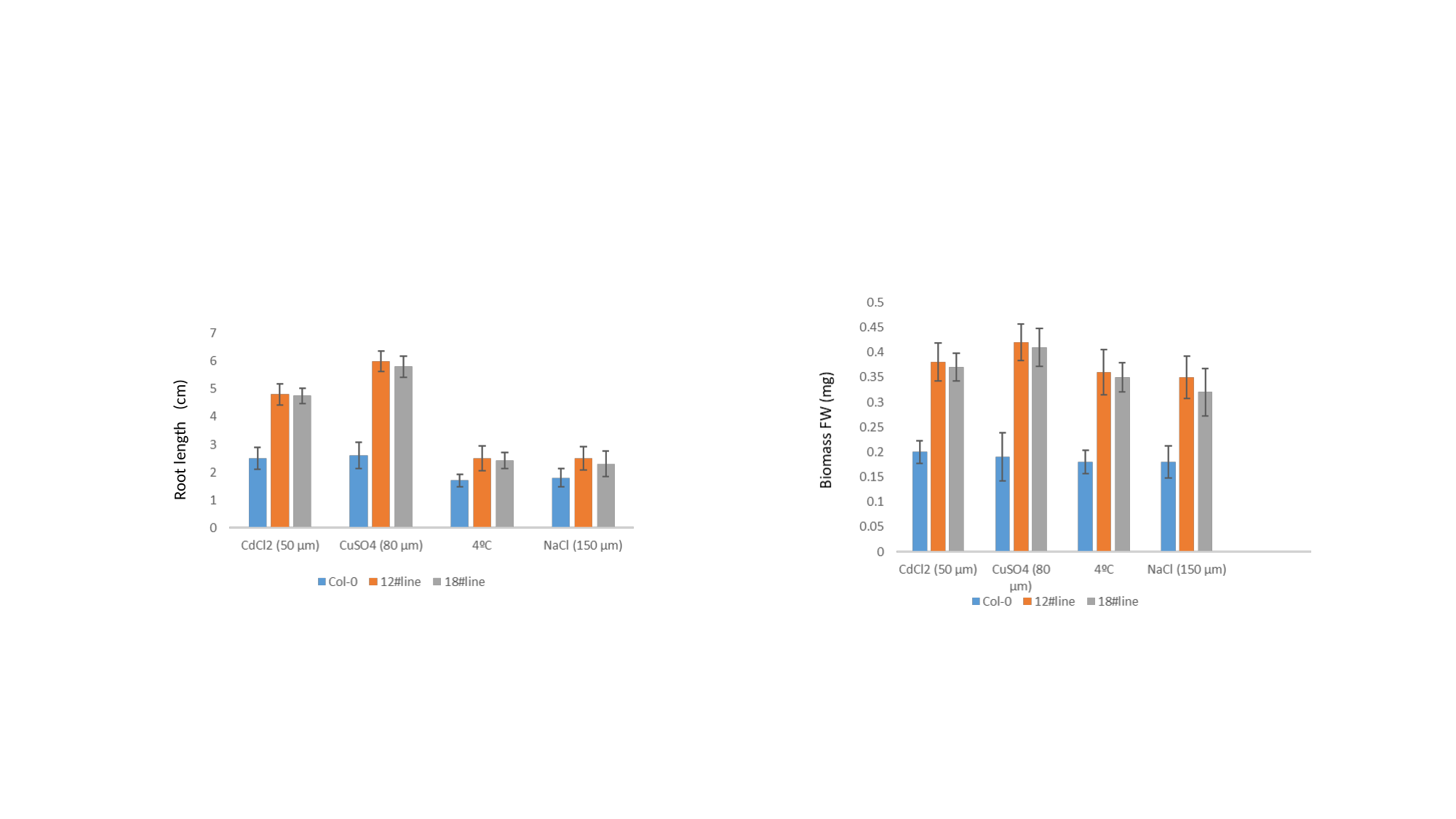

Root length (cm)

Supplement: Supplementary file 4 — Additional file 4: Fig. S4. Various abiotic stress conditions imposed to control vs overexpressed lines and their root length and biomass data. Values are means and bars indicate SDs (n=6) statistically significant difference at P< 0.05 (t-test). [file 43141_2022_457_MOESM4_ESM.ppt]
